# Early Vascular Aging in Children With Type 1 Diabetes and Ambulatory Normotension

**DOI:** 10.3389/fped.2021.764004

**Published:** 2021-12-20

**Authors:** Terezie Šuláková, Jiří Strnadel, Jan Pavlíček, Radka Poláková, Tomáš Seeman, Janusz Feber

**Affiliations:** ^1^Department of Pediatrics, University Hospital Ostrava, Ostrava, Czechia; ^2^Medical Faculty University of Ostrava, Ostrava, Czechia; ^3^Centre of Excellence IT4Innovations, Institute for Research and Applications of Fuzzy Modeling, University of Ostrava, Ostrava, Czechia; ^4^Department of Pediatrics, University Hospital Motol, Prague, Czechia; ^5^Second Medical Faculty Charles University, Prague, Czechia; ^6^Department of Pediatrics, Dr. von Hauner Children's Hospital, Ludwig Maximillians University, Munich, Germany; ^7^Children's Hospital of Eastern Ontario, University of Ottawa, Ottawa, ON, Canada

**Keywords:** ambulatory blood pressure monitoring (ABPM), arterial stiffness, children, diabetes type 1, early vascular aging

## Abstract

**Background:** Preliminary data suggest that target organ damage (TOD) and early vascular aging (EVA) may occur in children with normal blood pressure (BP).

**Objectives:** To analyze TOD and EVA in normotensive (BP <95th percentile on ambulatory BP monitoring) type 1 diabetes children (T1D) in comparison to healthy controls (C).

**Subjects:** 25 T1D aged 13.9 ± 2.6 years and 22 C aged 14.0 ± 3.4 years.

**Methods:** We analyzed age- and height-related pulse wave velocity (PWV) Z-scores and expected PWV based on age, height, and mean arterial pressure (MAP). Expected vascular age based on measured PWV was calculated from pooled pediatric and adult PWV norms. Left ventricular mass index (LVMI), estimated glomerular filtration rate (eGFR), and urinary albumin/creatinine ratio (ACR) were obtained as markers of TOD.

**Results:** T1D and C groups did not differ in anthropometry, ambulatory, LVMI, and ACR. However, median age- and height-related PWV Z-scores were higher in T1D compared to C (1.08 vs. 0.57, *p* = 0.006; 0.78 vs. 0.36, *p* = 0.02, respectively). Mean (±SD) difference between measured and expected PWV was 0.58 ± 0.57 in T1D vs. 0.22 ± 0.59 in C, *p* = 0.02. The mean (±SD) difference between chronological and expected vascular age was 7.53 ± 7.74 years in T1D vs. 2.78 ± 7.01 years in C, *p* = 0.04.

**Conclusion:** Increased arterial stiffness and increased intraindividual differences between expected and measured PWV as well as between chronological and expected vascular age indicate that EVA may develop in T1D children even at normal ambulatory BP levels.

## Introduction

Elevated blood pressure (BP) represents an important cardiovascular risk factor with a direct relationship between the BP level and rates of stroke, myocardial infarction, and the risk of end-stage renal disease (1).

In children, the BP cutoffs for increased cardiovascular risk are not clearly defined due to the lack of longitudinal studies linking childhood BP levels to long-term outcomes in adulthood (2). However, there is a high probability that a hypertensive child would become a hypertensive adult, a well-known phenomenon called BP tracking (3). The risk of CV complications is low during childhood, but children can develop target organ damage (TOD) as a consequence of untreated hypertension (4, 5).

Recent studies suggest that even mild elevation of BP, below the hypertension threshold, or white coat hypertension (WCH) can lead to heart and vascular damage (6–8). In children with kidney disease and/or diabetes mellitus, the vascular injury is further potentiated by the underlying disease, leading to a much higher risk of hypertension-related TOD (9, 10).

Pulse-wave velocity (PWV) can be used for non-invasive assessment of vascular function (i.e., arterial stiffness) and evaluation of vascular aging; it can be successfully applied to children for whom there are age- and height-specific normative data (11–15). Increased PWV is considered an early marker of hypertension-related TOD and marker of early vascular aging (EVA) (16, 17).

The goal of our study was therefore to analyze BP and PWV in children with diabetes mellitus type 1 in comparison with healthy controls.

We hypothesized that diabetic ambulatory normotensive children would show functional cardiovascular changes (increased arterial stiffness) and a higher vascular age compared to healthy controls even in the absence of structural changes [increased left ventricular mass (LVM), microalbuminuria] and that the functional changes would occur with only mild elevation of the BP level.

## Methods

### Patients

All consecutive patients with type 1 diabetes who were referred for assessment of hypertension to Pediatric Nephrology & Hypertension Clinic, Department of Pediatrics, University Hospital Ostrava, Czechia, from January 2017 to December 2019 were enrolled in a prospective study. Out of a total of 29 patients enrolled, 25 patients with T1D were diagnosed with ambulatory normotension based on ambulatory BP monitoring (ABPM) criteria (see below), and they were included in the current study.

The control group consisted of 22 healthy children (12 boys) recruited from hospital co-workers' family members.

All subjects completed the office BP measurements, ABPM, echocardiography, and pulse wave velocity (PWV) measurements. No patient was treated with antihypertensive therapy at the time of the investigation. All patients had normal renal function without any significant proteinuria except for one diabetic patient with albuminuria (albumin-to-creatinine ratio, 13.4 mg/mmol). The demographics of both groups is shown in Table 1.

**Table 1:** Basic characteristic of both study groups.

**Parameter**	**T1D (*n* = 25)**	**C (*n* = 22)**	***p***
Gender (female/male)	13/12	10/12	NS
Age	13.9 ± 2.6	14.0 ± 3.4	NS
Height (cm)	159.9 ± 13.2	159.8 ± 14.7	NS
Height-SDS	−0.3 ± 0.9	−0.02 ± 1.3	NS
Weight (kg)	55.1 ± 20.0	48.9 ± 13.8	NS
Weight-SDS	0.3 ± 1.3	−0.1 ± 1.0	NS
BMI (kg/m^2^)	19.7 (18.2, 23.7)	18.5 (16.6, 20.4)	NS
BMI-SDS	0.4 (−0.3, 1.5)	−0.2 (−1.0, 0.6)	NS
Diabetes duration (years)	5.1 ± 2.9	-	ND
Obesity + overweight, n (%)	6 (24)	1 (4.6)	NS
T-Cholesterol (mmol/l)	4.4 ± 0.7	4.2 ± 0.6	NS
HDL-cholesterol (mmol/l)	1.6 (1.4, 1.7)	1.3 (1.2, 1.5)	NS
LDL-cholesterol (mmol/l)	2.6 ± 0.5	2.7 ± 0.4	NS
Triglycerides	0.9 (0.7, 1.3)	0.9 (0.7, 1.1)	NS
HbA1C (mmol/mol) /n, %/	67.0 ± 7.4/8.3 ± 2.8/	-	ND
eGFR/creatinine (ml/s/1.73 m^2^)	1.8 (1.6, 2.1)	1.6 (1.4, 1.7)	0.005
eGFR/Cystatin C (ml/s/1.73 m^2^)	1.6 ± 0.2	1.5 ± 0.1	0.027
ACR (mg/mmol)	0.7 (0.4, 0.9)	0.5 (0.4, 0.7)	NS

*ACR, albumin/creatinine ratio; BMI, body mass index; eGFR, estimated glomerular filtration rate; NS, nonsignificant; ND, not done; SDS, standard deviation score. Data are expressed as mean ± SD or median [interquartile range (IQR)] as appropriate*.

Inclusion criteria were as follows: written informed consent and age 10–19 years. For children with diabetes type 1, the additional inclusion criterion was diabetes duration ≥1 year. The exclusion criteria were as follows: history of other serious disorders or history of diseases affecting BP (especially heart and kidney diseases), current antihypertensive medications, or other BP-affecting issues such drug abuse or smoking.

The study was conducted in accordance with the Declaration of Helsinki and was approved by the local ethics committee. Written informed consent was obtained from all parents and patients of both study groups, as appropriate.

### Anthropometric Measurement

At the time of the office and ABPM measurements, the body height and weight were recorded. The body mass index (BMI) was calculated as kg/m^2^; BMI, weight, and height were converted into standard deviation scores (SDS), e.g., Z scores, based on reference values for healthy Czech children; see http://www.ojrech.cz/lesny/kompendium/index.htm. Weight and height were measured by a single trained nurse with precision electronic scales and fixed stadiometer.

### Office Blood Pressure Measurement

The office BP was measured by a single trained nurse on the same day as the ABPM (before initiating the ABPM device), according to the current European guidelines (9). After 10 min of rest, the BP measurement was done with an automatic oscillometric Omron 705IT device^1^ (OMRON Healthcare Europe B.V., Hertogenbosch, Netherlands). The oscillometric device was validated for BP measurement in children^2^ The measurements were taken using the right arm, in the sitting position with the elbow at the level of the right atrium, using one of three cuff sizes (child: 6–12, medium: 12–23, or adults: 17–38.6 cm). The appropriate cuff size was determined by measuring the mid-arm circumference and was ~40% of the arm circumference (an inflatable bladder width). The first BP reading was used for analysis. The obtained absolute systolic BP (SBP) and diastolic BP (DBP) values were subsequently converted into Z-scores (SDS) based on age- and height-related normative values for children.

### Twenty-Four-Hour Ambulatory Blood Pressure Monitoring

The ABPM was performed using the oscillometric device SpaceLabs 90217 (SpaceLabs Medical Inc., Redmond, Washington, USA). The monitor was programmed to measure the BP every 20 min during the day (6 A.M.−10 P.M.) and every 30 min during the night (10 P.M.−6 A.M.). The parents and children were instructed to keep a diary of daily activities during the ABPM measurement. However, in order to compare our results with the normative values for ABPM (18), we defined the daytime period as 8 A.M.−8 P.M. (12 h) and the nighttime period as 12 P.M.−6 A.M. (6 h). The cuff size was determined by measuring the mid-arm circumference and was ~40% of the arm circumference. In all patients, the length of the cuff covered 100% of the arm circumference. The cuff was placed on the non-dominant arm. The patients were instructed to avoid vigorous physical exercise during the ABPM measurement but to follow their usual daily activities. A minimum of 40 ABPM recordings were required to consider the ABPM valid.

For the study purposes, the following ABPM parameters were obtained and analyzed: mean arterial pressure (MAP), SBP, and DBP measured over 24 h, daytime and nighttime periods. The average absolute values for MAP, SBP, and DBP for all time periods were subsequently converted into Z-scores (SDS) using the ABPM normative data (18). Night-time BP dipping was calculated using the ratio of mean daytime/mean nighttime MAP, SBP, and DBP. Non-dipping (absence of nocturnal BP fall at least 10%) was defined as day/night (D/N) ratio <1.1 in MAP and/or SBP and/or DBP. ABPM raw data were also used to estimate the ambulatory arterial stiffness index (AASI), which was calculated as 1 minus the regression slope of DBP on SBP values over 24-h period (19).

### Definition of Ambulatory Blood Pressure Monitoring Normotension

Normotension on ABPM was defined as mean SBP and DBP and MAP <95th percentile (i.e., <1.645 SDS) during 24 h, daytime and nighttime periods. Hypertension on ABPM was defined as mean SBP or DBP or MAP value ≥95th percentile (i.e., ≥1.645 SDS) at any time period. Similarly to adult and European Society of Hypertension (ESH) guidelines, the BP load was not included in the definition of ABPM hypertension (9).

### Definition of White Coat Hypertension

Patients with ABPM normotension (as defined above) and office SBP or DBP SDS >1.645 were categorized as patients with WCH.

### Arterial Stiffness

Arterial stiffness was assessed by carotid femoral pulse wave velocity (cfPWV) *via* applanation tonometry using validated PulsePen device (DiaTecne s.r.l.) (16, 20) as described previously (11, 20). All measurements were performed by one trained physician.

Prior to cfPWV measurement, patients were placed in the supine position and rested for 15 min in quite temperature-comfortable room. Three electrocardiographic leads were attached. The right carotid artery was palpated and marked (proximal site), and the procedure was repeated for the right femoral artery (distal site). To assess pulse wave travel distance, surface tape measurements were performed between the carotid site and the jugular notch and between the jugular notch and the femoral site. The difference between these two distances was considered the pulse travel distance (11, 16). PWV was examined by sequential recordings of the arterial pressure wave at the carotid and femoral arteries and was defined as the distance of the sampling sites divided by the time difference between the rise delay of the distal and proximal pulses according to the R wave belonging to the ECG QRS complex calculated by the software. The pulse wave was calibrated by measuring the BP immediately before each recording. The measurement of transit time was discarded and repeated if BP and HR varied by >10%, the variability between consecutive systolic or diastolic waveforms was >10%, and/or when the amplitude of the pulse wave signal was 80 mV. All of PWV measurements were performed 3–4 times in each participant, and the average of the two lowest PWV measurements with inter-measurement divergence ≤ 0.5 m/s was taken in analysis. The absolute PWV values were subsequently converted into age- and height-adjusted Z-scores (SDS) using normative pediatric data, which were obtained using the same PWV device (PulsePen) (11).

### Expected Pulse Wave Velocity and Vascular Age Calculation

To calculate the expected PWV in each child based on age, height, and MAP, we used the equation proposed by Reusz et al. (11) expressed as follows:


$$\begin{array}{l} “PWV\left(m/s\right)=0.049\times age\left(years\right)+0.008\times height\left(cm\right)\\ +0.024\times MAP\left(mmHg\right)+1.129.^{\prime \prime } \end{array}$$

The expected PWV results were then compared with obtained PWV results (PWV difference) in each individual patient and subsequently compared between T1D and C groups.

To assess vascular age of our children, we combined normative age-based PWV data for children aged 7–18 years (11) obtained with applanation tonometry device (PulsePen) with normative age-based data for adults aged 19–40 years (21) obtained with two different devices (SphygmoCor and Complior) for PWV measurement using applanation tonometry for males and females separately. There was a significant linear relationship between chronological age (in years) and 50th percentile PWV (m/s) across the whole chronological age range from 7 to 40 years: PWV in males (m/s) = 3.99 + 0.08 × age (years), *r*^2^ = 0.95, *p* <0.0001; PWV in females (m/s) = 4.01 + 0.08 × age (years), *r*^2^ = 0.96, *p* < 0.0001. The expected age based on measured PWV (vascular age) for individual patients was then calculated as follows: patient's measured PWV – 3.99 for males (4.01 for females)/0.08. The intraindividual differences between the chronological age and the vascular age were then compared between groups.

### Echocardiography

Echocardiography was performed using a General Electric Vivid 9 and 95e (General Electric, Milwaukee, WI) ultrasound systems. Measurements were performed off-line by a single experienced physician according to the guidelines of the American Society of Echocardiography (22). Two-dimensional echocardiography images were obtained for the analysis of left ventricular (LV) volumes on three consecutive beats from apical four- and two-chamber views. Wall thickness and chamber dimensions are obtained from the two-dimensional parasternal long axis or M-mode short axis at the midventricular level. The LVM was calculated by using the Devereux Equation (23) and indexed to the height^2.7^ [left ventricular mass index (LVMI)]. From the study purposes, LVMI was expressed as LVMI ratio, which was obtained by dividing the measured LVMI value by the 95th percentile of LVMI for healthy children (24). LVH was defined as LVMI ratio >1.0 (>95th percentile).

### Laboratory Parameters

All the laboratory investigations were performed on the day of ABPM. Blood draws of the patients were performed in the morning after overnight fasting. Biochemical analysis in whole blood [glycated hemoglobin (HbA1C)], serum (creatinine, lipid profile, cystatin C), and the first morning urine (albumin and creatinine) was measured by routine laboratory methods immediately after collection. These blood samples except HbA1C were centrifuged at 2,500 g for 6 min at 4°C. Serum concentration of total cholesterol (T-cholesterol), high-density lipoprotein cholesterol (HDL-cholesterol), low-density lipoprotein cholesterol (LDL-cholesterol), and triglycerides were measured by enzymatic methods on AU5420 analyzer (Beckman Coulter, Inc., USA). HbA1C was measured using high-performance liquid chromatography (HPLC; Tosoh G8, Tosoh Bioscience, Inc., CA, USA). The blood was collected in ethylenediaminetetraacetic acid (EDTA) anticoagulant tubes.

Serum and urine concentrations of creatinine were determined by the enzymatic method on AU5420 analyzer (Beckman Coulter, Inc., USA). Serum concentration of cystatin C and urine albumin was measured by immunonephelometric method (BN ProSpec, Siemens Healthcare, USA). The estimated glomerular filtration rate for creatinine (eGFR/creatinine) was calculated using the updated Schwartz formula (25). We also calculated eGFR based on the cystatin C equation developed by the Chronic Kidney Disease Work Group (26). The albumin/creatinine ratio (ACR) was analyzed from a first morning urine sample. Pathological albuminuria was defined as ACR >2.2 mg/mmol.

### Statistical Analysis

The distribution of continuous data was analyzed with the d'Agostino & Pearson omnibus test, normally distributed data are presented as mean ± SD, non-normally distributed data are shown as median and interquartile range (IQR; i.e., 25th and 75th percentile). Continuous variables were compared using Student's unpaired *T*-test (if normally distributed data) or Mann–Whitney test (if non-normally distributed data). In addition to the classic null hypothesis significance testing (NHST), we used estimation statistics with permutation on 5,000 resamples; results are shown as mean difference between groups, bias-corrected and accelerated confidence intervals, and permutation *p*-values. The magnitude of the difference between groups was estimated using Cohen's D effect size with bias-corrected accelerated 95% confidence intervals (27). Thresholds for Cohen D effect size include 0.2 (small effect), 0.5 (medium effect), and 0.8 (large effect). The categorical variables (proportion of patients between groups) were compared using a chi-square test or the Fisher's exact test.

The relationship between PWV, diabetes, and BP was analyzed using linear regression analysis. We also performed multivariate regression analysis with only selected, deemed as most clinically important, variables (*n* = 3) as predictors of PWV (dependent variable).

Results were considered statistically significant if the *p*-value was below 0.05. Statistics were performed using the GraphPad software, version 6.0, for Windows and Python (Jupyter Lab, package dabest version 0.3.1) (27).

## Results

### Patient Characteristics

Basic characteristics of T1D and healthy controls (C) are presented in Table 1. All children were of Caucasian origin. The average (± SD) duration of diabetes was 5.1 ± 2.9 years in T1D group. There were no significant differences between T1D and C patients in age, gender, anthropometric parameters, or proportion of obesity. There were also no differences in metabolic parameters such as T-cholesterol, HDL- and LDL-cholesterol, triglycerides, and ACR between both groups. However, the Schwartz eGFR and cystatin C eGFR was significantly higher in T1D group, most likely due to hyperfiltration (Table 1).

### Blood Pressure

The absolute and SDS values of office SBP and DBP were significantly higher in diabetic children compared to healthy controls (Table 2). However, ABPM (absolute and SDS) parameters of 24 h, day and night SBP, DBP, and MAP did not differ between both groups except for absolute day MAP (Table 2). There was also no difference in BP dipping. The proportions of patients with non-dipping hypertension or WCH were not significantly different between groups (Table 2). There were also no differences in pulse pressure and AASI.

**Table 2:** Blood pressure values.

	**T1D (*n* = 25)**	**C (*n* = 22)**	***p*-value**
Office SBP-SDS	0.9 ± 1.1	0.07 ± 1.1	0.006
Office DBP-SDS	0.2 ± 1.5	−1.0 ± 1.3	0.004
24-h SBP-SDS	−0.4 ± 0.8	−0.5 ± 0.8	NS
24-h DBP-SDS	−0.2 ± 0.7	−0.5 ± 0.9	NS
24-h MAP - SDS	0.2 ± 0.7	−0.2 ± 0.7	NS
Day SBP-SDS	−0.5 ± 0.8	−0.3 ± 0.9	NS
Day DBP-SDS	−0.3 ± 0.8	−0.4 ± 0.9	NS
Day MAP-SDS	0.1 (−0.7, 0.4)	−0.2 (−0.8, 0.6)	NS
Night SBP-SDS	−0.1 ± 0.7	−0.4 ± 0.8	NS
Night DBP-SDS	0.1 ± 0.8	−0.3 ± 0.8	NS
Night MAP-SDS	0.3 ± 0.6	−0.0 ± 0.6	NS
Number of people with WCH, n (%)	7 (28)	1 (5)	NS
SBP D/N	1.1 ± 0.1	1.2 ± 0.1	NS
DBP D/N	1.3 ± 0.1	1.3 ± 0.1	NS
MAP D/N	1.2 (1.1, 1.3)	1.2 (1.1, 1.2)	NS
Number of people with non-dipping, n (%)	11 (44)	6 (27)	NS
24-h PP	44.1 ± 5.2	45.0 ± 5.5	NS
24-h AASI	0.27 ± 0.1	0.25 ± 0.1	NS

*AASI, ambulatory arterial stiffness index; DBP, diastolic blood pressure; HR, heart rate; MAP, mean arterial pressure; n, number; NS, nonsignificant; PP, pulse pressure; SBP, systolic blood pressure; SDS, standard deviation score. Data are expressed as mean ± SD or median [interquartile range (IQR)] as appropriate*.

### Echocardiography

We did not find any significant differences between diabetic children and healthy controls in LVM, LVMI, and LVMI/95th percentile [median (25th, 75th percentiles): T1D 72.60 (62.45; 105.50) vs. C 70.80 (53.35; 100.20), T1D 22.00 (18.48; 25.00) vs. C 20.40 (18.25; 25.80), and T1D 0.52 (0.46; 0.59) vs. 0.50 (0.46; 0.59), respectively].

### Arterial Stiffness and Left Ventricular Mass

**Figure 1:**
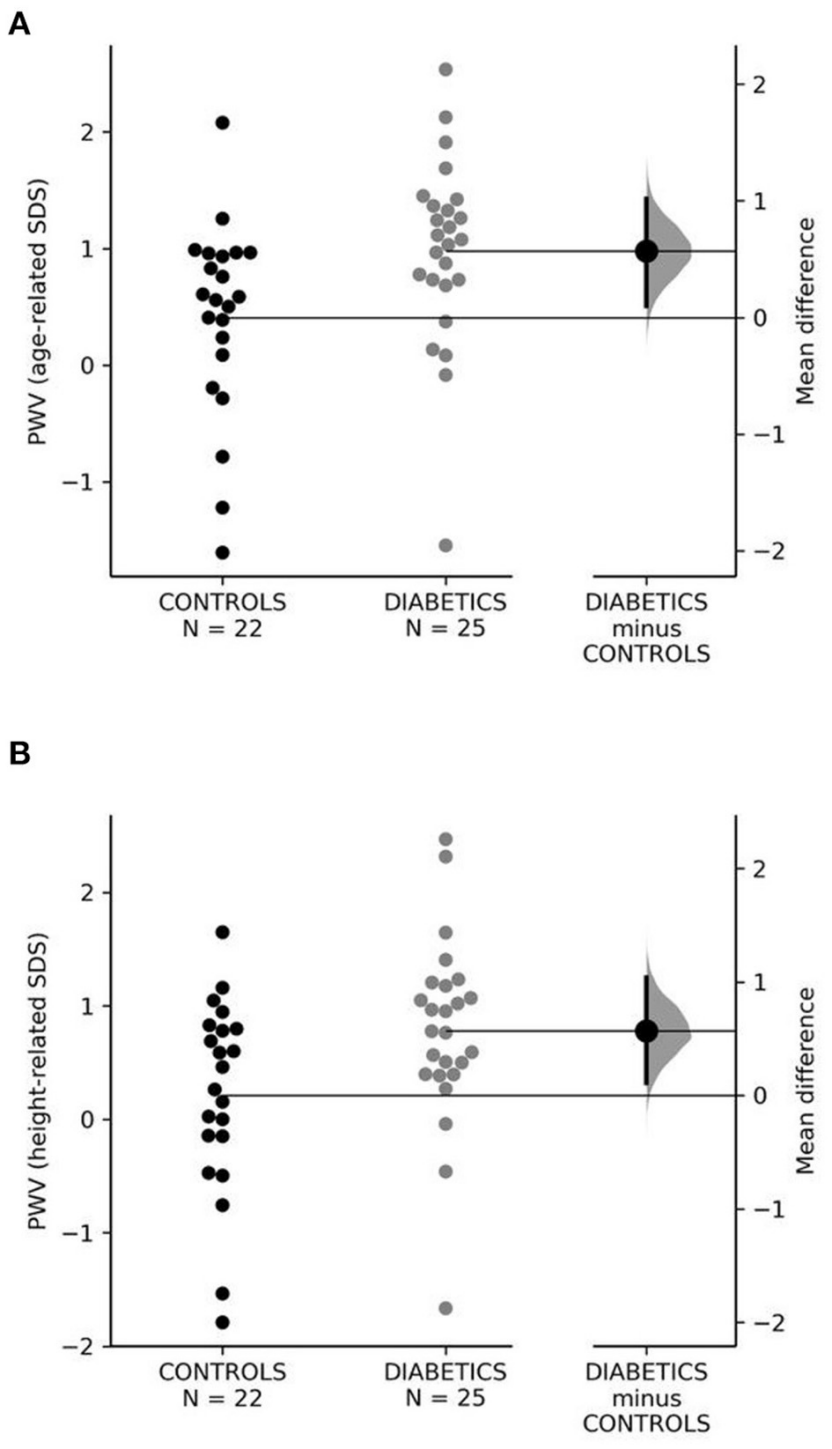
**(A,B)** Age- and height-related SDS of PWV in control and diabetic groups. PWV, pulse wave velocity; SDS, standard deviation score.

Children with diabetes type 1 had higher absolute values of PWV (m/s) (*p* = 0.037) and significantly higher age-related PWV SDS on standard NHST comparison (Table 3) and on estimation statistics: mean difference = 0.57; 95% CI = 0.1–1.02, permutation *p* = 0.02; Cohen's D effect size = 0.69, 95% CI = 0.00–1.24 (Figure 1A). Height-related PWV SDS was also significantly higher in T1D (Table 3): mean difference = 0.57; 95% CI = 0.11–1.04, permutation *p* = 0.03; Cohen's D effect size = 0.68, 95% CI = 0.02–1.22 (Figure 1B).

**Table 3:** Vascular parameters.

**Parameters**	**T1D**	**C**	***p*-value**
Measured PWV (m/s)	5.48 (5.25; 6.05)	5.16 (4.85; 5.84)	0.037
Measured PWV-SDS_age_	1.08 (0.71; 1.39)	0.57 (0.02; 0.96)	0.006
Measured PWV-SDS_height_	0.78 (0.39; 1.19)	0.36 (−0.23; 0.81)	0.022
Measured vs. predicted PWV difference (m/s)	0.58 ± 0.57	0.22 ± 0.59	0.020
Predicted vascular vs. chronological age difference (years)	7.53 ± 7.74	2.78 ± 7.01	0.04

*C, controls; PWV, pulse wave velocity; SDS, standard deviation score; T1D, diabetes type 1. Data are expressed as mean ± SD or median [interquartile range (IQR)] as appropriate*.

**Figure 2:**
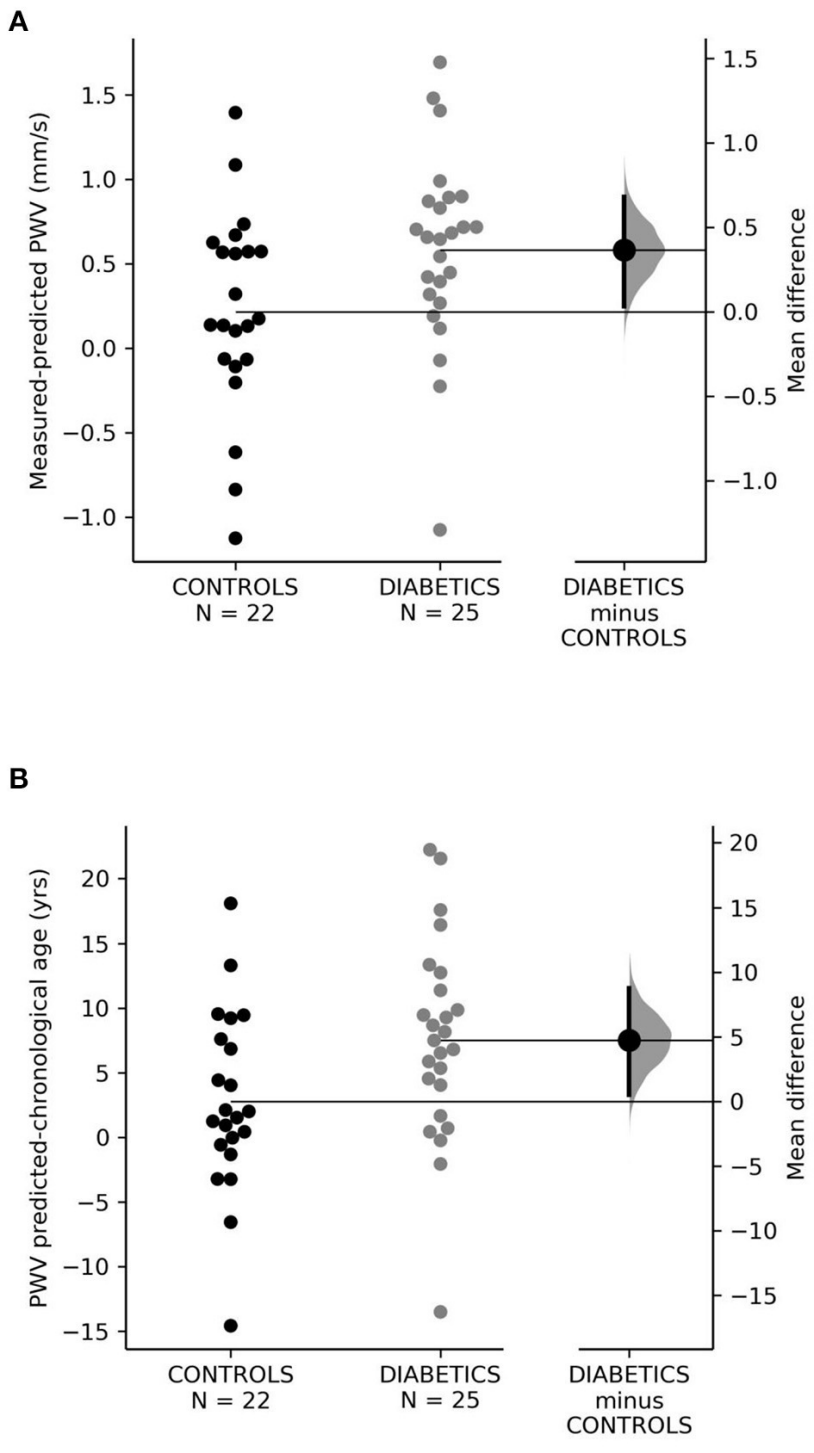
**(A,B)** Vascular age. yrs, years.

Using the formula for PWV prediction based on age, height, and MAP published by Reusz et al. (11), the PWV difference (measured vs. expected PWV) was significantly higher in T1D patients compared to controls (Table 3) with the mean difference of 0.36 m/s, 95% CI = 0.03–0.68, permutation *p* = 0.04; Cohen's D effect size was 0.63; 95% CI = 0.05–1.18 (Figure 2A).

In children with diabetes type 1, the difference between the PWV expected and actual chronological age was significantly higher in T1D patients as compared to the control group (Table 3); the mean difference was 4.75 years, 95% CI = 0.51–8.75, permutation *p* = 0.04, Cohen D effect size was 0.64 (95% CI = 0.02–1.2) (Figure 2B).

The comparison of LVM and LVMI did not show any significant differences between the groups (Table 3).

**Figure 3:**
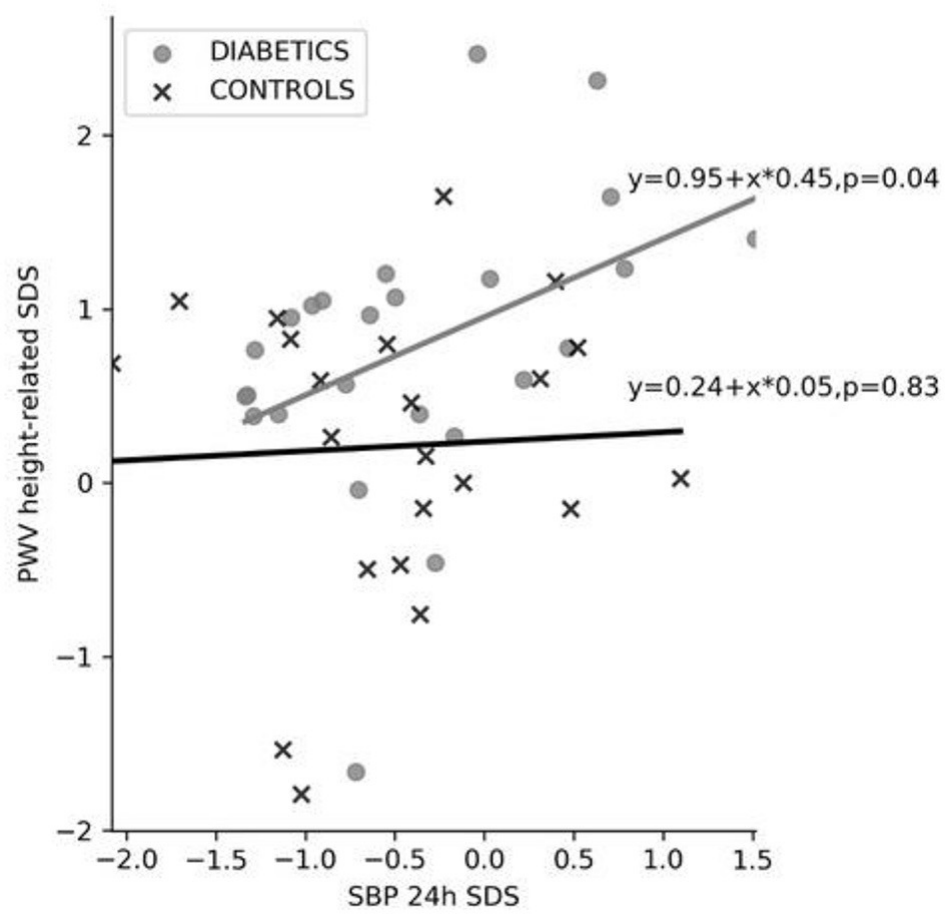
Relationship between 24-h SBP SDS and height-related PWV SDS. PWV, pulse wave velocity; SBP 24-h SDS, 24-h systolic blood pressure standard deviation score.

The relationship between 24-h SBP SDS and PWV height-related SDS is shown in Figure 3. While there is no significant correlation between of 24-h SBP SDS and PWV SDS (height-related) in controls, the correlation was significant (*r* = 0.42, *p* = 0.04) in diabetic children (Figure 3). Diabetic children also had a higher intercept and steeper slope of the regression line compared to controls (Figure 3).

A limited multivariate analysis (due to the low number of patients) including age, presence/absence of diabetes, and 24-h SBP showed age and diabetes as significant predictors of PWV (*r*^2^ = 0.39, *F* = 9.327, *p* < 0.0001) (Table 4A). In children with diabetes type 1, the 24-h SBP SDS and HbA1C value were significant predictors of PWV with a good overall correlation coefficient (*r*^2^ = 0.372, *F* = 6.520, *p* = 0.006) (Table 4B).

**Table 4:** Multivariate analysis.

**Names**	**coef**	**SE**	**T**	***p*-value**	***R* ^**2**^**	**Adjusted *R*^**2**^**	**97.5% CI**	**Relimp**	**Relimp %**
**(A) Model 1 for all children**.
Intercept	1.29	1.40	0.92	0.363	0.39	0.35	−1.54; 4.11	N/A	N/A
Age	0.10	0.03	3.15	0.003	0.39	0.35	0.04; 0.17	0.21	52.39
Diabetes	0.39	0.17	2.32	0.025	0.39	0.35	0.05; 0.72	0.08	19.45
24-h SBP	0.02	0.01	1.63	0.111	0.39	0.35	−0.01; −0.01	0.11	28.17
**(B) Model 2 for T1D only**.
Intercept	8.61	1.04	8.31	0.000	0.37	0.32	6.46; 10.75	N/A	N/A
24-h SBP-SDS	0.35	0.15	2.37	0.027	0.37	0.32	0.04; 0.66	0.16	42.32
HbA1C	−0.04	0.02	−2.76	0.011	0.37	0.32	−0.07; −0.01	0.22	57.68

*(A) Model 1: F-statistic = 9.327, p (F-statistic) < 0.0001*.

*(B) Model 2: F-statistic = 6.520, p (F-statistic) = 0.006*.

*HbA1C, current glycated hemoglobin; 24-h SBP, 24-h systolic blood pressure; SDS, standard deviation score*.

## Discussion

Our study showed that ABPM normotensive diabetic children and adolescents had significantly increased absolute PWV as well as age- and height-related PWV SDS compared to their normotensive controls. Moreover, the intraindividual differences between measured and predicted PWV were significantly higher in T1D patients compared to healthy controls. Similarly, children with diabetes type 1 had significantly higher intraindividual differences between chronological and PWV-predicted vascular age as compared to controls, suggesting early vascular aging (EVA) in T1D patients. This is a novel finding, not previously described. There was no difference in LVM index and microalbuminuria between patients with diabetes type 1 and healthy controls.

Diabetes mellitus has been proven to be a major risk factor for the development of cardiovascular disease (CVD) (28). Recent ESH guidelines (9) and some studies have consequently pointed out that children with diabetes type 1 have increased prevalence of hypertension (29) and are at higher risk of an early-onset CVD (10, 30–33). Children and adolescents with diabetes type 1 generally do not have manifest clinical signs of CVD. They may however suffer from a subclinical cardiac and vascular damage that can occur at BP levels that are even below the hypertension threshold (7). In pediatrics, the most frequently used indirect and non-invasive subclinical CVD markers are increased PWV and LVM.

PWV is the most widely accepted method for assessment of arterial stiffness and vascular age (34, 35) in both children and adults. In adults, PWV is related mainly to age and BP; further determinants are male gender and diabetes (36). Similarly to adults, children's PWV increases with age and is also dependent on sex (11–15). Furthermore, at the age of 18 years, the 50th (10th, 90th) percentiles of PWV appear to merge into reference values obtained in adults with optimal BP levels (12, 21).

In adults with diabetes type 1, the increased PWV is associated with adverse cardiovascular outcomes and increased likelihood of early cardiovascular morbidity and with all-cause and cardiovascular mortality, often in connection with various comorbidities (hypertension, chronic kidney disease, etc.) (31–33). Importantly, according to recent data from observational studies, the CV morbidity and mortality are seen in young adults and are associated with a shorter life span of 9.4–17.7 years of life (31). Comorbidities such as hyperglycemia, hypertension, dyslipidemia, diabetic kidney disease, insulin resistance, and obesity are still the strongest risk factors for CVD and mortality in type 1 diabetes.

The extent of cardiovascular morbidity in children with diabetes type 1 is not well-understood. However, many children and adolescents with diabetes type 1 fail to meet the American Diabetes Association (ADA) and International Society for Pediatric and Adolescent Diabetes (ISPAD) targets for HbA1C, SBP and DBP, LDL-cholesterol, and triglycerides (37), and many youth with diabetes type 1 are not treated or undertreated for hypertension, dyslipidemia, and microalbuminuria. They may therefore be at risk for CVD complications even in the absence of comorbidities and obvious clinical signs of CV injury. An early identification of subclinical CV injury may help with management of diabetes type 1 and other contributing factors, mainly arterial hypertension and proteinuria.

While the diagnosis of hypertension and its various forms (white coat, masked, true hypertension) and proteinuria (urine albumin/creatinine) is well-established and used by most physicians, the assessment of subclinical CV injury is still not routinely done in all children with diabetes type 1. Most centers use echocardiography to assess LVM, but the assessment of vascular stiffness using PWV is done mainly for research purposes.

There are several studies describing PWV (applanation tonometry) in children and adolescents with diabetes type 1. The recent meta-analysis (38), which included four age-matched case-control PWV studies with 1,491 children with a mean age of 15.2 years (975 patients with diabetes type 1 average duration of 7.1 years and 516 controls), showed significantly increased carotid–femoral PWV (absolute) values (m/s) in diabetic children compared with controls. The significant determinants of PWV in diabetic children were diabetes duration, age, and presence of diabetes; other important variables were gender and MAP.

In the prospective study with a 5-year follow-up (38), achievement of office BP <90th percentile for age, sex, and height was associated with significantly lower PWV during the follow-up (5.5 vs. 5.7 m/s, *p* = 0.04). Another recent study (39) including 1,809 youth with diabetes type 1 found an association of PWV mainly with diabetes duration and HbA1C but also with other determinants—adiposity, higher (office) BP, and adverse lipid levels, i.e., traditional CV risk factors. In none of the studies was ABPM used.

Our study is in agreement with the abovementioned studies. We found significantly increased arterial stiffness in ABPM-normotensive diabetic children compared with controls. Age, presence of diabetes type 1, and 24-h SBP were the strongest predictors of PWV in the whole group (Table 4A); in children with diabetes type 1, the predictors of PWV were the 24-h SBP SDS and HbA1C (Table 4B). These findings are similar to previously published studies (38–40). In most of the studies (except for two recent studies), BP and PWV were expressed in absolute values only. In contrast, we show all BPs and particularly PWV in absolute values and sex-, age-, or height-related Z-scores (SDS). This allowed for a more detailed quantification of observed results and direct comparison with sex- and age-/height-specific normative data. Moreover, most other studies (39, 41, 42) [except for (5)] used office BP for correlation with PWV, whereas we used ABPM 24-h BP, which is in general a better predictor of CV risk than office BP. Although we measured the office BP in our children, we did not consider it as a valid assessment of BP in our study population given the high variability of office BP, white coat effect, etc. Indeed, 28% of our ABPM normotensive diabetic children had an elevated office BP suggesting WCH. Although all diabetic children had normal BP on ABPM (as per inclusion criteria) (Table 2), the ABPM BP Z-scores were slightly/non-significantly increased (mean ± 24-h SBP and DBP Z-scores = −0.4/-0.2 SDS) (Table 2) compared to the control group (-0.5/-0.5 SDS). This mild increase of 24-h BP, albeit clinically not noticeable, significantly contributed to the increase of PWV/arterial stiffness in the presence of diabetes type 1, as suggested by the multivariate analysis (Table 3).

Because PWV increases with age, increased values of arterial stiffness in children can lead to premature vascular injury and EVA. Children with diabetes type 1 would be theoretically at a higher risk of EVA due to the cumulation of EVA risk factors (diabetes type 1 + hypertension). Our results confirmed our hypothesis that children with diabetes type 1 had higher intraindividual differences between chronological and predicted vascular age (Figure 2), suggesting an accelerated vascular aging as compared to healthy normotensive children. Our results show that children with diabetes type 1 suffer from EVA, which can be considered a novel finding, not previously described in pediatric literature. Children with EVA may be at an increased risk of future cardiovascular complications later in life, as observed in recent observational studies (31–33).

We also measured LVM in our study population and found no difference between children with T1D and control group. This is different from some other authors who found various abnormalities in LV geometry, LVM, and LV function (43, 44). The discrepancy between our and literature results may be due to the differences in BP levels, duration of diabetes type 1, indexation of LVM, use of normative values, and the fact that the healthy children may have a physiologically higher LVM due to physical activity regardless of the BP level. It would be reasonable to assume that morphological/structural LVM changes occur later in the course of the disease and at a higher BP level, whereas all our children with diabetes type 1 were normotensive on ABPM. While some minor/functional changes on echocardiography such as diastolic dysfunction can be expected and were described in children with diabetes type 1 (42–44), we did not measure diastolic function on echocardiography due to technical limitations in our institution.

### Limitations and Strengths/Advantages

Firstly, the number of study subjects is limited. However, we compared the results of diabetic children with a control group of healthy children using absolute values as well as sex-, age-, and height-related reference values (Z-scores). In addition, we used estimation statistics with permutation *p*-values and effect size estimation, which allowed us to draw conclusions from differences derived from a relatively small sample but estimated and confirmed on a large permutated sample (*n* = 5,000). The use of modern statistical estimation methods can be considered a strength of our study. Secondly, in our study, we used a different applanation tonometry device (PulsePen) than most other studies in diabetics (SphygmoCor). However, the obtained PWV results from both devices are comparable, with excellent concordance between these two devices (45). Moreover, the normative data for children were generated by the same device as the one used in our study (PulsePen). Thus, the use of applanation tonometry can be considered another strength of our study. Thirdly, as already discussed above, we did not measure the diastolic function on echocardiography.

## Conclusion

In conclusion, children with diabetes type 1 and ambulatory normotension have significantly increased arterial stiffness and PWV-predicted vascular age (EVA) while having normal LVM and no significant albuminuria. The main predictors of increased vascular stiffness in children with diabetes type 1 were the HbA1C levels and systolic 24-h BP. It appears that even a small increase in ambulatory BP, even within the normal range, can contribute to the development of vascular injury and EVA in the presence of diabetes type 1.

## Data Availability Statement

The original contributions presented in the study are included in the article/Supplementary Material, further inquiries can be directed to the corresponding author.

## Ethics Statement

The studies involving human participants were reviewed and approved by Ethics Committee, University Hospital Ostrava, 17. Listopadu 1790/5 708 52 Ostrava-Poruba e-mail: eticka.komise@fno.cz. Written informed consent to participate in this study was provided by the participants' legal guardian/next of kin.

## Author Contributions

TŠu: author of the supporting grant, responsible for the data collection, controls selection, office BP measurement, ABPM, PWV measurement, calculation of Z-scores, descriptive statistics, NHST, and writing a manuscript. JS: office BP measurement, diabetic patients selection, blood, and urine samplings. JP: echocardiography. RP: multivariate analysis. TSe: author of the supporting grant and revisions of manuscript. JF: revisions, including final version of the manuscript, statistic (NHST, estimation statistics with permutation), figures, calculation of vascular age, scientific leading, and discussion. All authors contributed to the article and approved the submitted version.

## Funding

This work was supported by the Ministry of Health, Czech Republic–conceptual development of research organization FNOs/2019 (MH CZ-DRO–FNOs/2019).

## Conflict of Interest

The authors declare that the research was conducted in the absence of any commercial or financial relationships that could be construed as a potential conflict of interest.

## Publisher's Note

All claims expressed in this article are solely those of the authors and do not necessarily represent those of their affiliated organizations, or those of the publisher, the editors and the reviewers. Any product that may be evaluated in this article, or claim that may be made by its manufacturer, is not guaranteed or endorsed by the publisher.

## Footnotes

^1^http://www.dableducational.org/sphygmomanometers/devices_1_clinical.html#ClinTable; and http://www.dableducational.org/pdfs/equivalence_declarations/E15%20Omron%20M4-I%20ESH-BHS.pdf

^2^ http://www.dableducational.org/accuracy_criteria.html
