# Supplementary material for: Early Vascular Aging in Children With Type 1 Diabetes and Ambulatory Normotension
Source: Front Pediatr. 2021 Dec 20;9:764004. doi: 10.3389/fped.2021.764004 (PMC8721847; doi:10.3389/fped.2021.764004)
Supplement: Supplementary file 1 [file Table_1.pdf]

Table S1 Absolute values of ambulatory blood pressure, heart rate and pulse pressure

	**T1D (n=25)**	**C (n=22)**	**P value**
office SBP (mmHg)	119.6±9.7	112.1±10.1	0.013
officeDBP (mmHg)	68.6±10.5	60.2±9.3	0.006
24hour SBP (mmHg)	110.3±7.0	109.6±6.5	NS
24hour DBP (mmHg)	66.1±3.9	64.6±4.8	NS
24hour MAP (mmHg)	81.8±4.6	80.5±4.3	NS
24hour HR (beat/minute)	83.2±8.9	78.6±10.2	NS
day SBP (mmHg)	114.1±7.3	115.1±7.5	NS
day DBP (mmHg)	70.7±4.7	70.5±5.7	NS
day MAP (mmHg)	86.9 (81.1, 89.7)	85.6 (81.6, 90.1)	0.013
day HR (beat/minute)	88.5±9.3	84.6 ±11.7	NS
night SBP (mmHg)	100.6±6.9	98.7±6.6	NS
night DBP (mmHg)	55.8±4.5	53.8±4.8	NS
night MAP (mmHg)	72.8±4.7	71.0±3.8	NS
night HR (beat/minute)	70.3±10.2	66.8 ±10.0	NS
day PP	43.4±5.4	44.7±6.1	NS
night PP	44.8±6.5	44.9±5.7	NS

Legend: DBP – diastolic blood pressure, HR – heart rate, MAP – mean arterial pressure, n – number, NS – non-significant. PP – pulse pressure, SBP – systolic blood pressure
